# Dysregulation of CD39/Ectonucleoside Triphosphate Diphosphohydrolase 1 Causes Urinary Bladder Dysfunction with Abnormal Smooth Muscle Contractility

**DOI:** 10.1096/fj.202504341RRR

**Published:** 2026-03-27

**Authors:** Zhaobo Luo, Huan Chen, Ali Wu, Weipu Mao, Sagar Barge, Seth L. Alper, Simon C. Robson, Weiqun Yu

**Affiliations:** ^1^ Department of Medicine, Beth Israel Deaconess Medical Center Harvard Medical School Boston MA USA; ^2^ Department of Anesthesia, Beth Israel Deaconess Medical Center Harvard Medical School Boston MA USA

**Keywords:** bladder dysfunction, ectonucleoside triphosphate diphosphohydrolases, lower urinary tract symptoms, purinergic signaling, smooth muscle contractility

## Abstract

Lower urinary tract symptoms (LUTS) are prevalent among the aging population, and current medications offer limited efficacy. Improved therapies require a better understanding of the mechanisms underlying LUTS. Abnormal extracellular ATP levels and altered purinergic contractility have been reported in patients with LUTS, suggesting dysregulation of the purinergic pathways. CD39/ENTPD1 is a major regulator of purinergic metabolism, and patients with ENTPD1 loss‐of‐function mutations exhibit bladder hypomotility and incontinence, suggesting critical roles for ENTPD1 in bladder function. We have tested this hypothesis in an overexpression mouse model with human *ENTPD1* knock‐in (*hCD39TG*), in a hemizygous *Entpd1* mouse model (*Entpd1* ^*+/−*^) mimicking LUTS patients with impaired ATP hydrolysis, and in a homozygous *Entpd1*‐deleted mouse model (*Entpd1* ^*−/−*^) mimicking the complete ENTPD1 loss‐of‐function observed in patients. We have demonstrated that ENTPD1 dysregulation leads to profound abnormalities in bladder voiding phenotypes and urodynamics, along with impaired BSM contractility and altered purinergic receptor signaling. Furthermore, we have found that modulation of downstream P2Y12/adenosine A2b receptor signaling partially or fully restores normal bladder function. Finally, we have identified expression of ENTPD1 and associated purinergic proteins in human BSM cells, underscoring the critical role of ENTPD1 in human bladder function and highlighting its translational potential for the treatment of LUTS.

## Graphical abstract

Abnormal extracellular ATP levels cause lower urinary tract symptoms (LUTS). Here, we demonstrate that overexpression of ENTPD1 (*CD39TG*)—an ATP/ADP converting enzyme—reduces voiding frequency while increasing void volume. Conversely, ENTPD1 deficiency results in frequent, small‐volume voiding. This effect is mediated by downstream P2Y12 and adenosine A2b receptor signaling. Furthermore, pharmacological modulation of these pathways rescues bladder dysfunction. These findings establish ENTPD1 as a master regulator of bladder function and highlight its translational potential for treating LUTS.

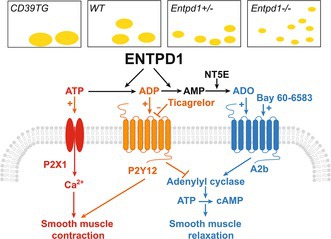

## 1 Introduction

The urinary bladder stores and periodically expels urine. Both processes require coordinated bladder smooth muscle (BSM) function. BSM cells relax and elongate to accommodate increasing urine volume during bladder filling (storage function). In contrast, BSM cells are activated to contract during bladder voiding, allowing complete urine expulsion (voiding function). Dysregulation of these processes leads to lower urinary tract symptoms (LUTS), which affect ~50% of the population over 40 years of age [1, 2]. Antimuscarinics and *β*3 adrenergic agonists are current first‐line drugs for LUTS treatment. However, these medications exhibit only marginal efficacy with high rates of adverse effects, often leading patients to discontinue treatment [3, 4]. Thus, a greater understanding of the mechanisms underlying LUTS and the development of novel therapeutics for LUTS remain significant unmet needs.

Regulation of bladder function by purinergic signaling involves a complex network including the extracellular signaling molecules ATP, ADP, and adenosine, which activate purinergic P2X, P2Y, and adenosine receptors [5, 6, 7, 8]. Extracellular signaling by ATP, ADP, and adenosine is tightly modulated by multiple enzymes, including CD39/ectonucleoside triphosphate diphosphohydrolases (ENTPD), ecto‐nucleotide pyrophosphatase/phosphodiesterase (ENPP), ecto‐5′‐nucleotidase (NT5E), and alkaline phosphatase (ALPL) [9, 10, 11, 12, 13, 14]. In both human and rodent bladders, ATP and ADP stimulate BSM contraction, whereas adenosine relaxes BSM [15, 16, 17, 18, 19, 20, 21, 22]. Abnormal extracellular ATP levels [15, 16] and altered purinergic contractility [17, 18, 19] have been reported in LUTS patients. Patients with overactive or obstructed bladders exhibit impaired ATP hydrolysis, with bladder contractility disproportionately sensitive to neuromuscular purinergic signaling as compared to normal bladders, suggesting that modulation of extracellular purine kinetics plays important roles in both bladder physiology and pathophysiology [15, 16, 17, 18, 19, 20, 21].

We have previously defined multiple ENTPDs in the mouse bladder wall, with ENTPD1 predominantly expressed in BSM cells [13]. ENTPDs are key enzymes that sequentially convert extracellular ATP to ADP and then to AMP, thereby modulating the temporal and spatial availability of extracellular purinergic signaling molecules for their corresponding receptors. In the ENTPD1‐deleted mouse bladder, profound deficiency in purinergic metabolism is accompanied by abnormal purinergic contractility [23]. Our own observations consistently indicate that BSM from *Entpd1*‐null mice exhibit prolonged hyperactivity in response to exogenous ADP stimulation [24]. Human patients with ENTPD1 loss‐of‐function mutations display bladder hypomotility and incontinence [25]. These findings strongly suggest that ENTPD1 is a critical regulator of bladder purinergic metabolism, BSM contractility, and normal bladder function. However, the mechanistic understanding of ENTPD1's role in mouse bladder function, and the roles of ENTPD1 and associated pathways in human BSM, remain unclear. Here we demonstrate the essential role of ENTPD1 in regulating bladder contractility and voiding function using multiple transgenic mouse models, including a human transgenic *ENTPD1* knock‐in mouse model (*hCD39TG*) overexpressing human ENTPD1 [26], a hemizygous ENTPD1‐deleted mouse model (*Entpd1* ^*+/−*^) to model impaired ATP hydrolysis as might be noted in human LUTS, and a homozygous ENTPD1‐deleted mouse model (*Entpd1* ^*−/−*^) replicating complete loss of function mutations found in some human patients [27].

## 2 Methods and Materials

### 2.1 Reagents

Unless otherwise specified, all chemicals were from MilliporeSigma and of reagent grade or better. P2X1 receptor agonist *α*, *β*‐methyleneadenosine 5′‐triphosphate trisodium (*α*, *β*‐meATP, Catalog #: 3209), P2Y12 receptor antagonist ticagrelor (Catalog #: 6884), and adenosine A2b receptor agonist Bay 60–6583 (Catalog #: 4472) were from RandD Systems.

### 2.2 Sex as a Biological Variable

Our study examined both male and female animals, and largely similar findings are reported for both sexes, with subtle differences noted in voiding in females and males in the CD39/ENTPD1 transgenic mice.

### 2.3 Animals

All mice in this study were of C57BL/6J background and matched for sex and age (12–16 weeks). Both male and female mice were used unless otherwise specified. Mice were housed in standard polycarbonate cages and maintained on a 12:12‐h light–dark cycle at 25°C with free access to normal food and water. Wild‐type *C57BL/6J* mice were purchased from Jackson Laboratory (Bar Harbor, ME). *CD39TG*, *Entpd1* ^*+/−*^, and *Entpd1* ^*−/−*^ mice provided by Dr. Simon Robson were bred in the BIDMC animal research facility.

### 2.4 Nucleotidase Histochemistry

A lead phosphate method was used to determine bladder tissue activities of ATPase, ADPase, and AMPase as previously described [28]. Briefly, 8 μm tissue sections of 4% paraformaldehyde‐fixed male wild‐type, CD39TG, Entpd1^+/−^, and Entpd1^−/−^ mouse bladders were preincubated with Tris‐maleate sucrose (TMS) buffer containing 0.25 M sucrose, 50 mM tris maleate and 2 mM CaCl_2_ pH 7.4 for 30 min at room temperature. The enzymatic reactions were performed at 37°C for 60 min in TMS‐buffered substrate solutions [2 mM Pb (NO_3_)_2_, 5 mM MgCl_2_, 5 mM MnCl_2_, and 3% dextran T250 (Sigma‐Aldrich) containing 1 mM final concentrations of substrate: ATP, ADP or AMP (Sigma‐Aldrich)]. Control sections were incubated in the absence of substrate. Lead orthophosphate precipitated by nucleotidase activity was visualized as brown deposits by incubating sections in 1% (*v/v*) (NH_4_)_2_SO_3_ for 1 min. Sections were counterstained with hematoxylin and dehydrated in graded ethanol, mounted in Permount mounting medium (Fisher Scientific). Fixed, stained sections were imaged with an Olympus BX61 microscope. Staining intensity was quantified using ImageJ software (National Institutes of Health, Bethesda, MD, USA). Regions of interest (ROIs) were defined within anatomically matched areas of the muscle layer. Mean gray values were measured and optical density (OD) was calculated according to the formula OD = log₁₀ (255/mean gray value). For each group, measurements from 3 to 5 randomly selected fields were averaged and used for statistical analysis.

### 2.5 Void Spot Assay (VSA)

VSA was performed during daylight from ~9:00 to 13:00 in both male and female mice. Individual mice were gently placed in a standard mouse cage lined with Blicks Cosmos Blotting Paper (Catalog no. 10422–1005), without drinking water but with standard dry mouse chow available. Blotting paper was recovered and imaged as described [29]. Overlapping voiding spots were visually examined and manually separated by outlining and copying, then pasting to a nearby empty space in Fiji software for analysis. A volume: area standard curve defined a 1‐mm^2^ voiding spot as representing 0.283 μL of urine. Urine spots of area ≥ 80 mm^2^ were considered primary voiding spots (PVS) based on a cutoff established from the voiding spot patterns of hundreds of mice [29, 30].

### 2.6 Cystometrogram (CMG)

CMG performed in *wild‐type* female mice with PBS infusion (25 μL/min) as previously described produces a consistent voiding pattern [25, 31]. A 1‐cm midline abdominal incision was performed under urethane (1.4 g/kg body weight) and continuous‐flow isoflurane (3% induction, 1.0% maintenance) anesthesia. PE50 tubing was implanted through the dome of the bladder. The catheter was connected to a pressure transducer. A syringe pump was coupled to data‐acquisition devices (WPI Transbridge and AD Instruments PowerLab 4/35) and a computerized recording system (AD Instruments LabChart software). Isoflurane was withdrawn immediately after surgery and CMG was performed under urethane anesthesia, which spares the voiding reflex. CMG was performed for > 1 h of stable, regular voiding cycles. At least 5 cycles of filling and voiding traces were assessed to evaluate urodynamics. Bladder compliance was determined by $\frac{∆\text{volume}}{∆\text{pressure}}=\frac{∆t\;x\;v}{∆\text{pressure}},v=25\mathrm{\mu L}/\min$.

### 2.7 BSM Myography

BSM myography was performed on male mice, whose ~30 mg bladders (larger than in females) facilitate dissection of the bladder epithelial layer away from BSM for a consistent per‐bladder yield of 4 ~ 7 mm by ~2 mm mucosa‐free muscle strips. Muscle strips were mounted in an SI‐MB4 tissue bath system (World Precision Instruments, FL, USA). Force sensors were connected to a TBM 4 M transbridge (World Precision Instruments). Signals were amplified by PowerLab (AD Instruments, CO, USA) and monitored with Chart software (AD Instruments). BSM strips gently stretched to optimize contractile force and pre‐equilibrated ≥ 60 min were subjected to electrical field stimulation (EFS, Grass S48 field stimulator (Grass Technologies, RI, USA)) with 2000/s sampling (Chart software) using standard protocols (50 V; 0.05 ms; trains of stimuli duration 3 s; frequencies: 1, 2, 5, 10, 20, and 50 Hz) [25, 31].

### 2.8 Human Bladder Procurement

Anonymized male and female human bladder samples were procured by the National Disease Research Interchange (NDRI, Philadelphia, PA) under protocol # RYUW1 from deceased organ donors aged 18–60 years old. Organ donors were free of both malignant and benign urinary dysfunction history, including bladder cancer, spinal cord injury, benign prostatic hyperplasia, urinary retention, urinary tract infection, and diabetes mellitus. Procured bladders were submerged in UW/SPS‐1 or HTK preservation solution, maintained at 4°C on ice, and shipped to our laboratory by air within 24 h. The middle equatorial region bladder tissue was fixed in 4% (*w/v*) paraformaldehyde, cryoprotected, and frozen at −80°C. This procedure is not considered by NIH to “involve Human Subjects,” as defined by NIH Supplemental Instructions, Part II.

### 2.9 Masson's Trichrome Staining and Imaging

Excised bladders were fixed in 4% formaldehyde, embedded in paraffin blocks, and the middle equatorial region of the bladder was sectioned for staining with Masson trichrome for bladder morphology evaluation. Imaging was performed on an Olympus BX60 fluorescence microscope with a 40×/0.75 objective [31].

### 2.10 Immunofluorescence Staining and Imaging

Excised mouse bladders and human bladder tissue fixed in 4% (w/v) paraformaldehyde were cryoprotected, frozen, sectioned (5 μM), and incubated overnight at 4°C with antibodies (1:100, Table S2). The sections were then incubated with an Alexa Fluor 488–conjugated secondary antibody (diluted 1:100), and nuclei were stained with DAPI. Mounted sections were imaged on an Olympus BX60 fluorescence microscope with a 40×/0.75 objective [31].

### 2.11 Western Blot

Whole bladder tissues from female mice were lysed with RIPA buffer (50 mmol/L Tris–HCl, 150 mmol/L NaCl, 1% NP‐40, 0.5% sodium deoxycholate, and 0.1% SDS). Protein concentrations were determined by BCA protein assay (Thermo Scientific, Rockford, IL, USA). 25 μg protein per well was fractionated by SDS‐PAGE and transferred to PVDF membrane. Membranes were incubated with primary antibodies (Table S2) at 4°C for 12 h. Immunoreactive protein bands were visualized with Amersham ECL reagent (Arlington Heights, IL, USA). The membranes were incubated with Restore Plus Western Blot Stripping buffer (Thermo Fisher Scientific, Rockford, IL, USA) for 5 min to remove primary and secondary antibodies for reblotting with new antibodies, and each membrane was stripped and re‐probed for no more than three times. Scanned protein band intensity was quantitated using Fiji software [31]. The protein band intensity of interest was normalized to glyceraldehyde 3‐phosphate dehydrogenase (GAPDH) or *β*‐actin intensity in the same lane. The average protein expression level in wild‐type mice was designated as 1, and the fold change of protein expression level in transgenic mice was reported.

### 2.12 Statistical Analyses

All data are presented as box and whisker plots. The centerline in box and whisker plots is the median, the box represents 75% of the data, and the whiskers indicate the range from minimum to maximum. Data were analyzed by 1‐way ANOVA for comparison among groups. Data were analyzed by Student's *t*‐test between two groups. If possible, a paired *t*‐test was used. Bonferroni's multiple comparison post hoc tests were used where necessary, and *p* < 0.05 was considered significant.

### 2.13 Study Approval

All animal studies were performed per NIH guidelines for animal care and use, and with approval of the Beth Israel Deaconess Medical Center Institutional Animal Care and Use Committee (Protocol#: 007–2022).

## 3 Results

### 3.1 Differential ENTPD1 Levels Lead to Altered Nucleotidase Activities in the Mouse Bladder

**FIGURE 1:**
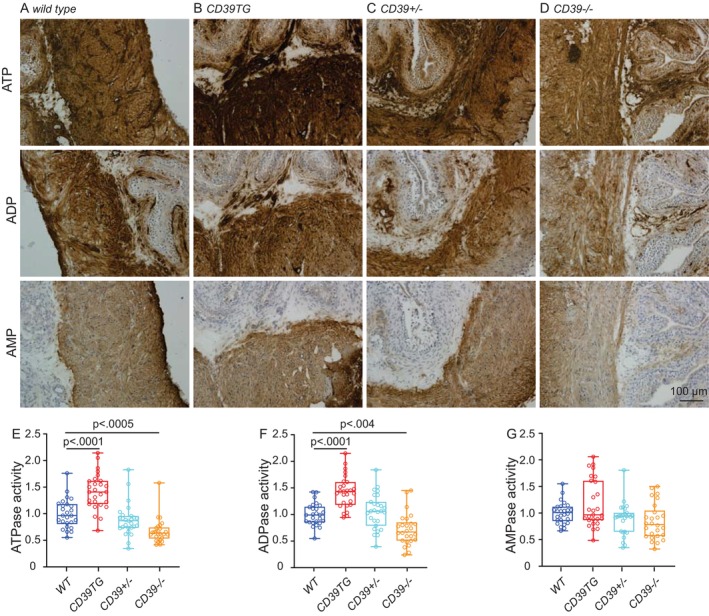
Differential ENTPD1 levels lead to altered nucleotidase activities in the mouse bladder. Representative images for nucleotidase histochemistry are shown from (A) wild‐type (*n* = 26 ROI from 3 females), (B) CD39TG (*n* = 26 ROI from 3 females), (C) Entpd1+/− (*n* = 26 ROI from 3 females), and (D) Entpd1−/− (*n* = 26 ROI from 3 females). (E–G) are quantified data. Data are plotted in Box (75% of the data) and whiskers format (range from minimum to maximum), with centerline as median value. Student *t*‐test, with *p* values above bars.

Enzyme histochemistry under Mn^2+^/Pb^2+^ conditions reflects the hydrolytic activity of tissues in response to exogenous nucleotides. Alterations in nucleotide hydrolytic activities were observed in the bladders of different *ENTPD1* genotypes. As expected, ATPase activity was significantly increased in the BSM of *CD39TG* mice, whereas a marked decrease in ATPase activity was detected in the BSM layer of *Entpd1* ^*−/−*^ bladder. Similarly, ADPase activity showed corresponding upregulation and downregulation in *CD39TG* and *Entpd1* ^*−/−*^ bladders (Figure 1). Although changes in AMPase activity did not reach statistical significance, a similar trend was observed. Taken together, these findings demonstrate marked alterations in nucleotide hydrolytic activities in the BSM layer.

### 3.2 ENTPD1 Expression Level Is Strongly Associated With Mouse Voiding Phenotype and Urodynamics

**FIGURE 2:**
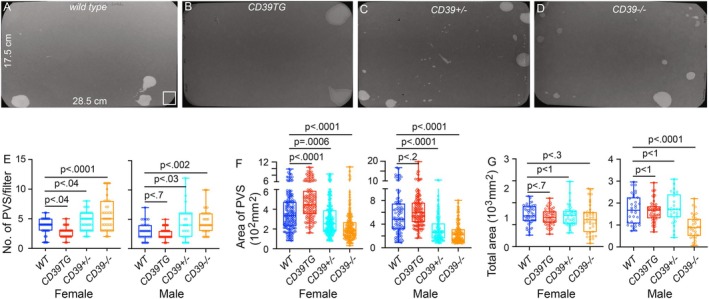
ENTPD1 expression level is strongly associated with the mouse voiding phenotype. Representative VSA filter images are shown from (A) wild‐type (*n* = 34 male, *n* = 48 female), (B) CD39TG (*n* = 56 male, *n* = 53 female), (C) Entpd1+/− (*n* = 28 male, *n* = 45 female) and (D) Entpd1−/− (*n* = 33 male, *n* = 38 female). (E–F) are quantified data: Primary voiding spot counts (PVS) are defined as voiding spots > 80mm^2^. Data are plotted in Box (75% of the data) and whiskers format (range from minimum to maximum), with centerline as median value. Student *t*‐test, with *p* values above bars.

**FIGURE 3:**
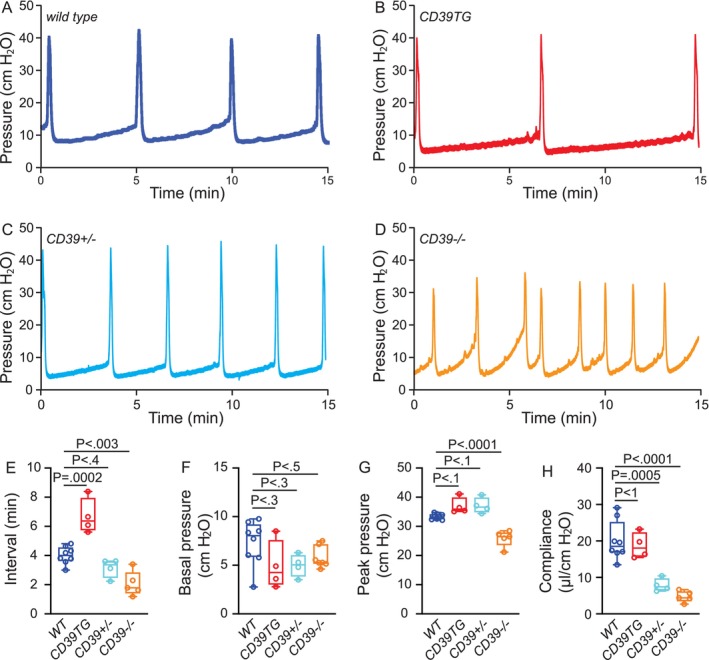
ENTPD1 expression level in mouse bladder is strongly associated with urodynamics. Representative CMG traces of (A) *wild‐type* (*n* = 8), (B) *CD39TG* (*n* = 4), (C) *Entpd1* ^*+/−*^ (*n* = 4), and (D) *Entpd1* ^*−/−*^ mice (*n* = 5) are quantified in (E–H). Data are plotted in Box (75% of the data) and whiskers format (minimum to maximum), with centerline as median value. Student *t*‐test, with *P* values above bars.

We performed the voiding spot assay (VSA) to examine whether changes in ENTPD1 expression directly affect the voiding phenotype in mice (Figure 2). Compared to wild‐type mice, female CD39TG mice produced larger primary voiding spots (PVS: spot area > 80 mm^2^) but significantly fewer numbers of PVS (Figure 2A,B,E,F). Male CD39TG mice exhibit similar trends, although the differences here were not statistically significant. In contrast, both *Entpd1* ^*+/−*^ and *Entpd1* ^*−/−*^ mice displayed significantly increased numbers of PVS with smaller PVS area than wild‐type mice of either sex (Figure 2A–F). These results indicate crucial roles for ENTPD1 in the regulation of both void frequency and void volume. Further cystometrogram (CMG) studies performed in female mice were consistent with the VSA findings. CD39TG mice exhibited significantly longer voiding intervals than wild‐type controls, whereas *Entpd1* ^*+/−*^ and *Entpd1* ^*−/−*^ mice showed markedly shorter voiding intervals (Figure 3). Additional analysis indicated decreased bladder compliance in ENTPD1‐deficient mice, with *Entpd1* ^*−/−*^ mice showing significantly reduced peak voiding pressure. These data corroborating the VSA results further demonstrate the essential role of ENTPD1 in the regulation of bladder contractility and urodynamics.

### 3.3 ENTPD1 Expression Is Crucial for Smooth Muscle Contractile Force

**FIGURE 4:**
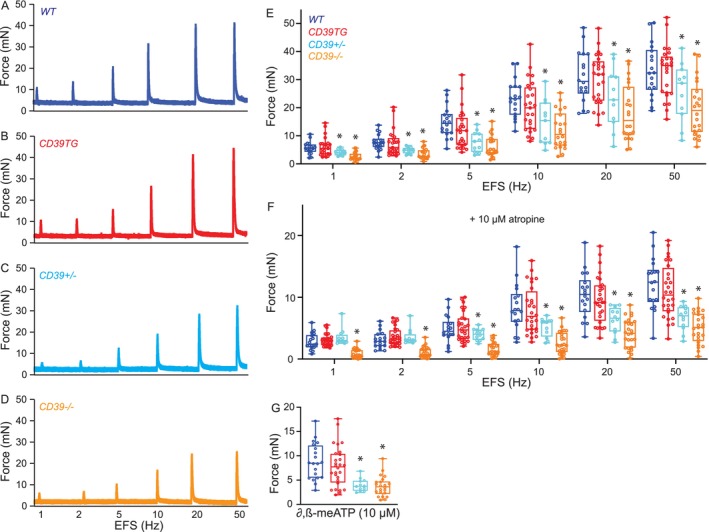
ENTPD1 expression is crucial for smooth muscle contraction force. Representative BSM contraction force traces in response to EFS are shown as (A) Blue: *Wild‐type*, *n* = 18; (B) Red: *CD39TG*, *n* = 25, (C) azure: *Entpd1* ^*+/−*^, *n* = 11, and (D) yellow: *Entpd1* ^*−/−*^, *n* = 22. (E–G): Summarized data from multiple experiments for BSM contractile force in response to EFS (E), atropine (F), and *α*, *β*‐meATP (G). Data are plotted in Box (75% of the data) and whiskers format (minimum to maximum), with centerline as median value. Student *t*‐test, with *indicate *p* ≤ 0.05 above bars.

BSM cells play a central role in bladder contraction and relaxation, and CMG data from *Entpd1* ^*−/−*^ mice suggested weakened BSM contraction force (Figure 3). Although previous studies demonstrated that *Entpd1* ^*−/−*^ BSM exhibit abnormal contraction responses to externally added nucleotides [23, 24], in vivo BSM contractility remains unstudied. To address this, we subjected freshly isolated BSM strips to myography (Figure 4 A–D). BSM strip contractile force increased in response to increased electrical field stimulation (EFS) frequencies, mimicking in vivo BSM contraction induced by EFS‐evoked neurotransmitter release. Contractile force of BSM strips from CD39TG mice was indistinguishable from that of wild‐type control strips. In contrast, BSM strips from ENTPD1‐deficient mice exhibited markedly reduced contractile force, with the most profound impairment in *Entpd1* ^*−/−*^ mice (Figure 4E). Wild‐type bladder contraction is mediated mainly by parasympathetic co‐release of ATP and Acetylcholine (ACh). These agents activate both purinergic P2X1 and muscarinic CHRM3 receptor‐mediated signaling cascades, leading to BSM contraction [25]. To evaluate possible changes in the signaling pathways in these mice, we tested the effect on BSM contraction of the CHRM3 receptor antagonist, atropine. As expected, atropine significantly inhibited EFS‐induced BSM contraction in wild‐type and CD39TG mice (Figure 4F), and the remaining contraction force (~40%) is largely purinergic. However, atropine produced far greater inhibition of EFS‐induced BSM contraction force in ENTPD1‐deficient mice (Figure 4F), indicating severely weakened purinergic contractility and suggesting possible alterations in bladder purinergic receptor expression and function. Further studies using the P2X1 receptor agonist, *α*, *β*‐meATP, confirmed minimal purinergic contractile responses in bladders from ENTPD1‐deficient mice (Figure 4G).

### 3.4 Alterations in ENTPD1 Expression Level Cause Distinct Mouse Bladder Morphological Changes

**FIGURE 5:**
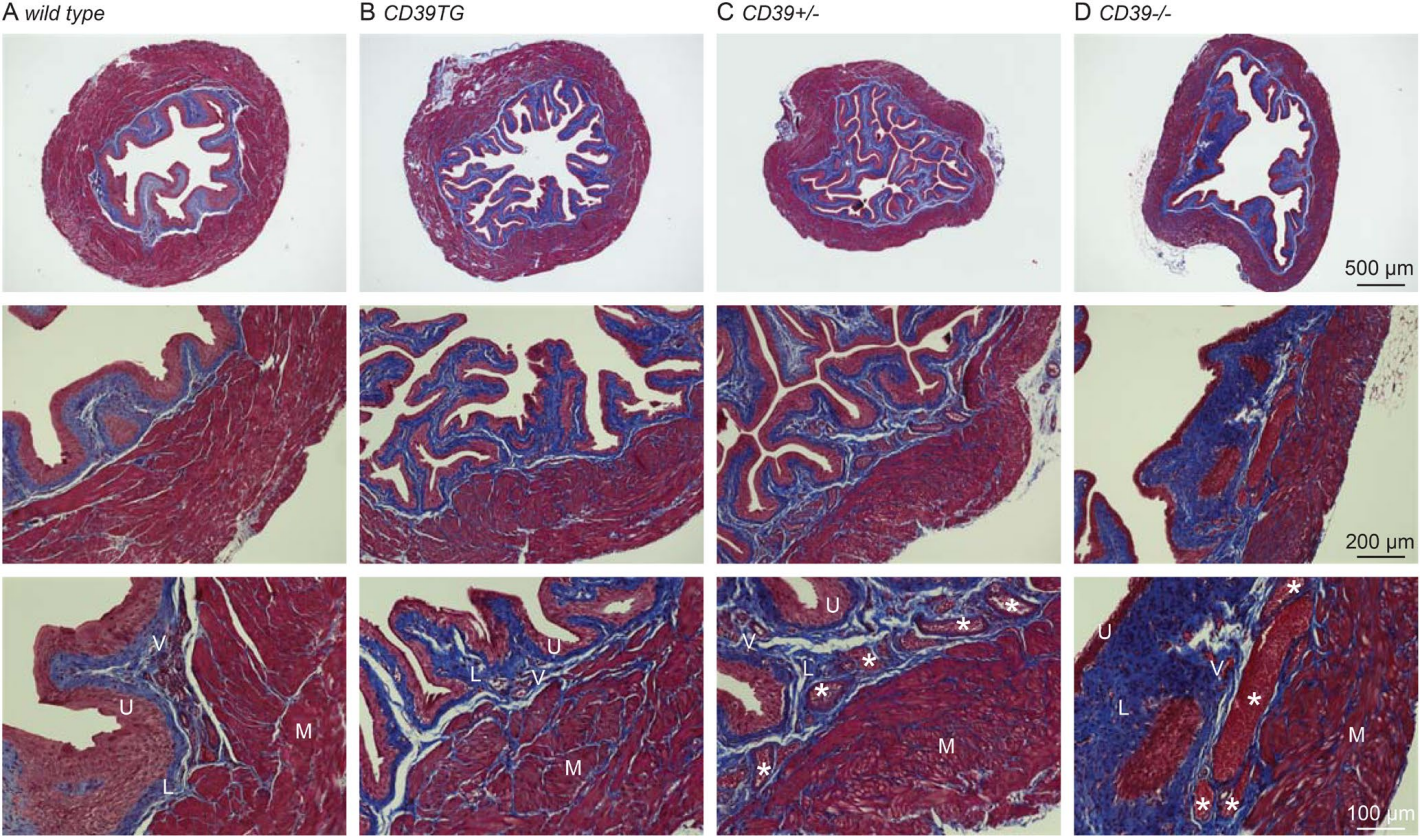
Alterations in ENTPD1 expression level cause distinct mouse bladder morphological changes. Representative images of (A) *wild‐type*, (B) *CD39TG*, (C) *Entpd1* ^*+/−*^, and (D) *Entpd1* ^*−/−*^ mice bladders. Enlarged images of bladder wall from panels *A*–*D* are shown below. L, lamina propria layer; M, smooth muscle layer; U, urothelium layer; V, blood vessel. White stars indicate dilated blood vessels in the lamina propria.

Alterations in ENTPD1 expression level did not affect mouse body weight or bladder weight (Table S1). However, Masson's trichrome staining of bladder sections revealed distinctive morphological changes (Figure 5). CD39TG mouse bladders exhibited increased urothelial ruffles, which may reflect increased bladder capacity, while the thickness of the BSM layer remained comparable to that of wild‐type controls. In contrast, bladders from *Entpd1* ^*+/−*^ and *Entpd1* ^*−/−*^ mice exhibited BSM layers markedly thinner than those of wild‐type controls (Table S2). Notably, increased urothelial ruffles were also evident in *Entpd1* ^*+/−*^ mouse bladders. In contrast, *Entpd1* ^*−/−*^ mouse bladders exhibited largely flattened urothelial surfaces accompanied by a thickened lamina propria layer, possibly indicative of potential fibrosis. Prominent in both *Entpd1* ^*+/−*^ and *Entpd1* ^*−/−*^ bladders was the presence of dilated vasculature, especially pronounced in *Entpd1* ^*−/−*^ mice. This pathological vascular alteration is consistent with both the dilated bladder phenotype and the impairment in BSM contraction force observed in ENTPD1‐deficient mice.

### 3.5 Differential Entpd1 Levels Provoke Altered Purinergic Signaling in the Mouse Bladder

**FIGURE 6:**
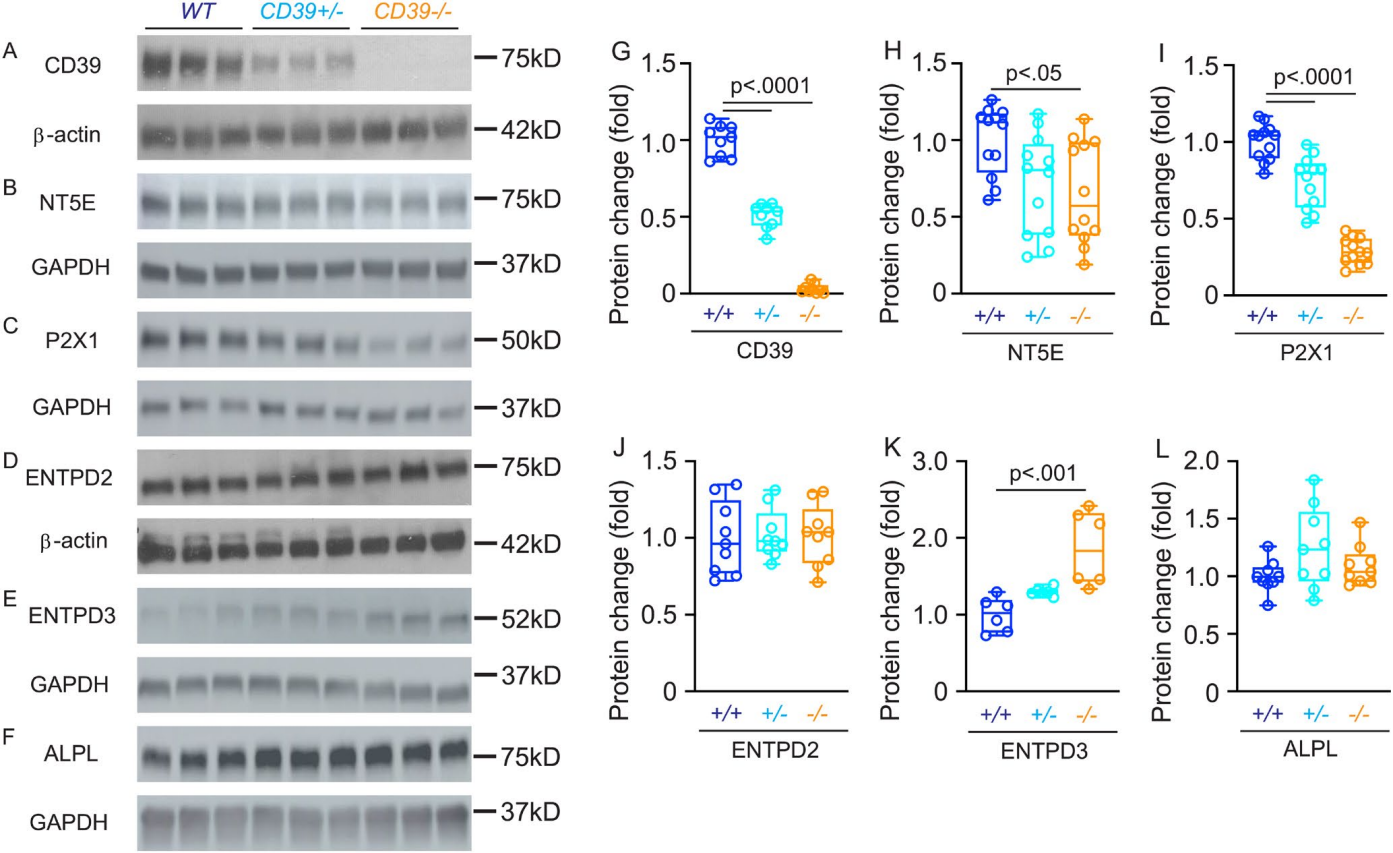
ENTPD1 Dysregulation causes altered purinergic signaling in the mouse bladder. (A–F): Western blots of ENTPD1 (*n* = 9), NT5E (*n* = 12), P2X1 (*n* = 12), ENTPD2 (*n* = 9), ENTPD3 (*n* = 6), and ALPL (*n* = 9) proteins from male *wild‐type, Entpd1* ^*+/−*^, and *Entpd1* ^*−/−*^ mouse bladders. *β*‐Actin and GAPDH were normalization controls for quantitated data in (G–L). Data are plotted in Box (75% of the data) and whiskers format (minimum to maximum), with centerline as median value. Student *t*‐test, *p* values above bars.

We extended our study of the mechanisms underlying the profound morphological and functional alterations observed in ENTPD1‐deficient mouse bladders with Western blot and immunofluorescence imaging studies. As shown in Figure 6A,G, ENTPD1 protein expression was completely absent in bladder samples from *Entpd1* ^*−/−*^ mice and significantly reduced in samples from *Entpd1* ^*+/−*^ mice. Interestingly, this deficiency was accompanied by a marked reduction in NT5E, a BSM enzyme that converts AMP to adenosine (Figure 6B,H) [28]. The ATP‐gated cation channel P2X1, responsible for triggering BSM contraction, was also significantly downregulated in ENTPD1‐deficient mouse bladder (Figure 6C,I). The bladder interstitial cell enzyme ENTPD2 [13] showed unchanged expression in ENTPD1‐deficient mice (Figure 6D,J). The reductions in expression of ENTPD1, NT5E, and P2X1 were evident in immunofluorescent images, with substantial loss of BSM membrane signal in *Entpd1* ^*+/−*^ mice, and yet further reduction or absence in *Entpd1* ^*−/−*^ mice (Figure S1). The profound alterations in *Entpd1* ^*−/−*^ bladder urothelial ruffles (Figure 5) prompted our examination of bladder expression of ENTPD3 and ALPL, two enzymes expressed in urothelial cells [14]. Notably, ENTPD3 expression was significantly increased in *Entpd1* ^*−/−*^ bladder (Figure 6E,F,K,L).

We next examined expression of these purinergic signaling components in the CD39TG mouse bladder. In contrast to our findings in ENTPD‐deficient mice, levels of ENTPD1, NT5E, ENTPD3, and P2X1 were comparable to those in wild‐type controls. However, ENTPD2 expression was significantly downregulated, whereas ALPL expression was significantly upregulated (Figure S2).

To determine whether dysregulation of ENTPD1 and associated purinergic signaling affects additional bladder cell phenotypes, we examined expression of muscarinic receptor M3 (CHRM3), a key contractile regulator of BSM. We also analyzed major smooth muscle differentiation markers, including *α*‐smooth muscle actin (*α*‐SMA), transgelin (SM22), and myosin heavy chain 11 (MYH11). None of these proteins differed significantly among bladders from *wild‐type*, *CD39TG*, *Entpd1* ^*+/−*^, and *Entpd1* ^*−/−*^ mice (Figures S3 and S4).

### 3.6 Human BSM Express ENTPD1 and Other Purinergic Signaling Molecules

**FIGURE 7:**
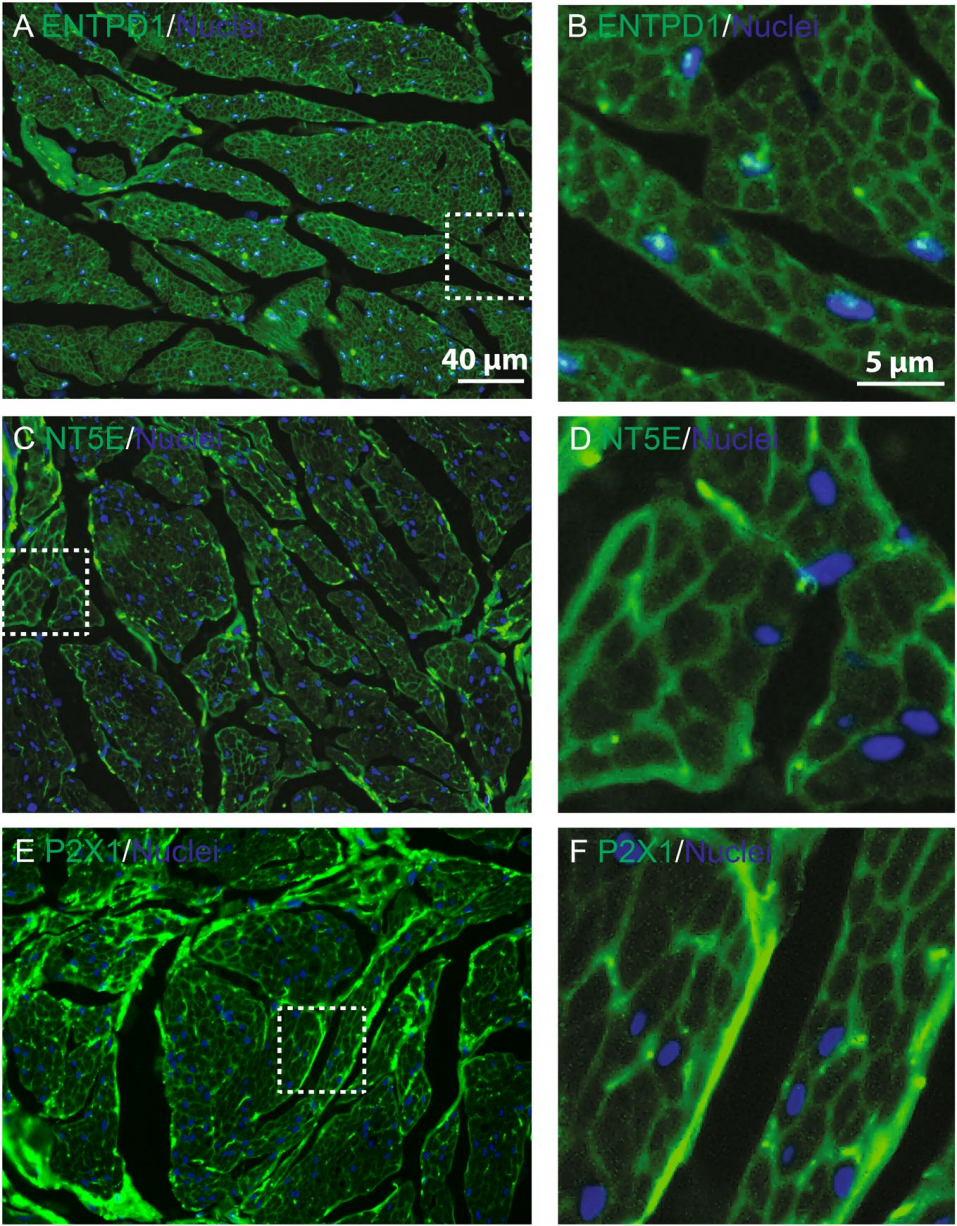
Human BSM express ENTPD1 and other purinergic signaling proteins. (A, C, and E): Human bladder tissues immunostained for ENTPD1, NT5E, and P2X1 (green), colocalized with DAPI‐stained nuclei (blue). Boxes with white dashed lines are enlarged in (B, D, and F), showing BSM membrane localization of these proteins. Scale bars are in white in panels (A) and (B).

ATP and ADP stimulate bladder contraction in both humans and mice, suggesting the presence of a purinergic regulatory system conserved across species. However, the specific characteristics of this system in the human bladder remain undefined. Our previous studies demonstrated the expression of ENTPD3 and ALPL in both human and mouse urothelial cells [14], supporting a similar expression pattern of purinergic signaling components in both human and mouse bladders. As expected, ENTPD1 (Figure 7A,B), as well as NT5E and P2X1 receptor (Figure 7C–F), are clearly detected on the human BSM cell membrane. These findings underscore the potential functional importance of ENTPD1 and associated purinergic pathways in regulating human bladder physiology and pathophysiology.

### 3.7 Bladder Dysfunction due to ENTPD1 Dysregulation Can Be Fully Rescued by Targeting Downstream Receptor Signaling

**FIGURE 8:**
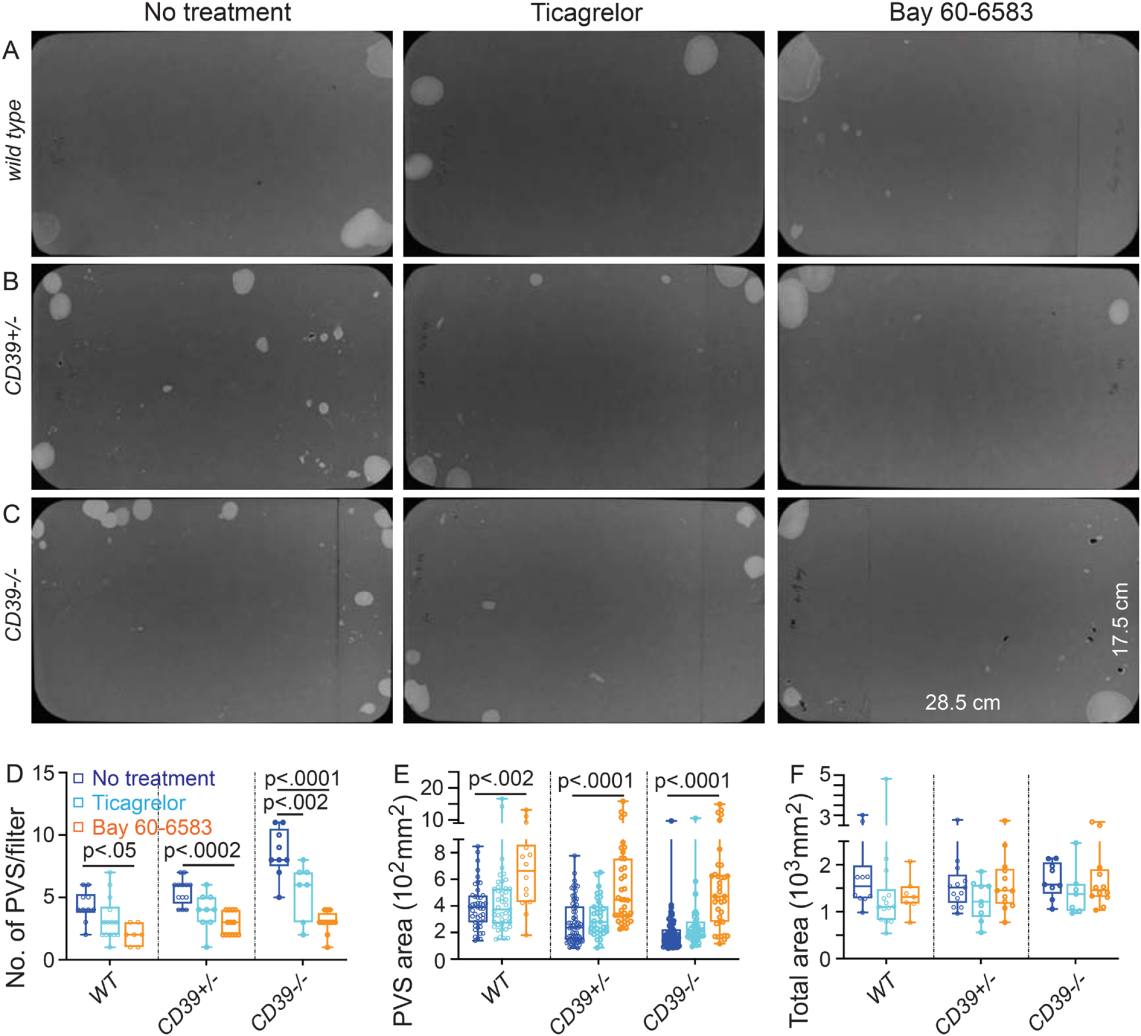
Bladder dysfunction due to ENTPD1 dysregulation can be fully rescued by targeting downstream receptor signaling. (A): Representative VSA filter images from female *wild‐type* (A: *N* = 7–14), *Entpd1* ^*+/−*^ (B: *N* = 9–13), and *Entpd1* ^*−/−*^ mice (C: *N* = 7–12) untreated (left panels) or treated with Ticagrelor (middle panels) or Bay 60–6583 (right panels). (D–F) show quantified data: Primary voiding spot counts (PVS, defined as voiding spots > 80mm^2^) are plotted in Box (75% of the data) and whisker format (minimum to maximum), with centerline as median value. Student *t*‐test, with *p* values above bars.

Lack of antibodies immunospecific for immunofluorescent imaging studies prevented our morphological demonstration of expression of P2Y12 and adenosine A2b receptors in human BSM cells. However, previous functional studies and single‐cell RNA sequencing analysis have provided strong evidence for human bladder expression of P2Y12 and A2b receptors [16, 20, 32, 33, 34]. We have shown previously that *P2Y12* knockout mice exhibit increased bladder capacity and reduced voiding frequency, whereas *A2b*‐KO mice have an overactive bladder (OAB) with decreased capacity and increased voiding frequency [25]. These findings suggest that P2Y12 and A2b receptors are key downstream targets regulated by ENTPD1, likely through modulation of extracellular nucleotide‐ and adenosine‐dependent kinetics in both human and mouse BSM cells. We then tested whether using selective agonists or antagonists targeting these receptors could rescue the abnormal bladder phenotypes observed in female *Entpd1* ^*+/−*^ and *Entpd1* ^*−/−*^ mice. Intraperitoneal injection of Ticagrelor, a selective P2Y12 antagonist, significantly reduced the PVS number in *Entpd1* ^*−/−*^ mice but did not increase the reduced PVS size. The data suggest partial rescue of bladder dysfunction in *Entpd1* ^*−/−*^ mice via inhibition of the P2Y12 receptor pathway (Figure 8). In contrast, activation of adenosine A2b receptor signaling by intraperitoneal injection of Bay 60–6583 completely restored normal bladder function in both *Entpd1* ^*+/−*^ and *Entpd1* ^*−/−*^ mice, as evidenced by dramatically reduced PVS numbers and increased PVS size (Figure 8). These results identify adenosine A2b receptor signaling as a critical downstream pathway responsible for bladder dysfunction associated with ENTPD1 dysregulation.

## 4 Discussion

ENTPD1 is the dominant ectonucleotidase, initially isolated from human endothelial cells, vascular smooth muscle cells, and immune cells by Robson and colleagues [35]. ENTPD1 has been implicated in a variety of physiological and pathological processes, including platelet aggregation, immunoregulation, protection against ischemic injury of the myocardium or kidney, and diabetes mellitus (DM) [36, 37, 38, 39, 40, 41]. Human *ENTPD1* single‐nucleotide polymorphisms (SNPs) have been identified that impact levels of expression, and genetic mutants that result in loss of function have been associated with multiple autoimmune diseases and with hereditary spastic paraplegias (HSP) [42, 43, 44, 45, 46, 47, 48]. The prominent and well‐documented bladder dysfunction that often accompanies these disorders suggests an important link between ENTPD1 and voiding dysfunction [49, 50]. Consistent with this, our data indicate a critical role of ENTPD1 and associated purinergic signaling in regulating bladder function by modulating BSM contractility, voiding frequency, and bladder capacity.

CD39TG mice, a transgenic mouse model overexpressing human CD39, exhibit 1.8‐ to 2.4‐fold higher catalytic activity of ENTPD1 [26]. In contrast, *Entpd1* ^*+/−*^ mice show reduced ENTPD1 activity with ~50% of wild‐type protein expression, and *Entpd1* ^*−/−*^ mice lack all ENTPD1 activity [23]. Consistently, our nucleotidase histochemistry data indicated altered nucleotidase activities in those mice (Figure 1). A striking observation of our study correlates higher ENTPD1 expression with lower voiding frequency and larger voids, whereas reduced or absent ENTPD1 expression results in increased voiding frequency and smaller voids (Figures 2 and 3). This direct relationship between ENTPD1 expression levels and bladder phenotype highlights the critical role of ENTPD1 and purinergic signaling in regulating bladder function.

Although ENTPD1 deficiency produces increased voiding frequencies and smaller voids, morphological analysis revealed a dilated bladder with expanded vasculature (Figure 5), suggesting normal or increased bladder capacity. This apparent paradox can be explained by decreased bladder compliance and lessened BSM contractile force (Figures 3 and 4), which likely reduces voiding efficiency, perhaps leading to increased residual urine or urinary retention. This reduced voiding efficacy decreases the bladder volume available for incremental urine accumulation, particularly in the presence of compromised bladder wall compliance. These conditions can generate the vicious cycle of BSM hypomotility, increased voiding frequency, small voided volumes, and a morphologically dilated bladder.

ENTPD expression and function in the human bladder remain poorly understood. In this study, we clearly demonstrated the presence of ENTPD1 and other purinergic system proteins in human BSM cell membranes (Figure 7), revealing an expression pattern similar to that of the mouse bladder. A recent proteomic study by the Lower Urinary Tract Dysfunction Research Network (LURN) identified ENTPD1 as the most highly upregulated serum protein in male human LUTS patients, with high elevation also noted in the urine of female LUTS patients [51]. However, ENTPD1 expression level in the local bladder tissue of those LUTS patients was not reported so it is not certain whether higher blood levels directly correlated with loss from endothelial cells and BSM cells and the release of ENTPD1 in exosomes [52]. Importantly, locally impaired ATP hydrolysis was recently reported in the human overactive bladder and/or obstructed bladder [53, 54, 55], suggesting dysregulated ENTPD1 function in LUTS. These findings highlight a potential strong association between ENTPD1 dysfunction and LUTS, although direct clinical evidence is lacking. Further studies are needed to clarify the role of ENTPD1 expression and function in LUTS pathogenesis in both diseased animal models and in human LUTS patients. Such investigations may uncover a predominant contribution of ENTPD1 to LUTS development, as demonstrated in our mouse models in this study, with potentially important clinical implications for the treatment of LUTS patients.

As a major regulatory enzyme for extracellular ATP and ADP metabolism, ENTPD1 profoundly impacts purinergic receptor expression and function in BSM cells (Figure 6 and Figure S1). This effect likely arises from dysregulated extracellular ATP/ADP/adenosine kinetics, which can lead to aberrant receptor expression and function. For example, due to ENTPD1 deficiency, excessive extracellular ATP quickly causes BSM P2X1 desensitization, endocytosis, and eventual degradation. Such receptor alterations could further exacerbate bladder dysfunction resulting from ENTPD1 dysregulation. Importantly, the bladder dysfunction of our Entpd1‐dysregulated mouse models was partially or fully rescued by targeting downstream P2Y12 and adenosine A2b receptor pathways. Notably, activation of adenosine A2b receptor signaling completely restored normal bladder function in our *Entpd1* ^*+/−*^ and *Entpd1* ^*−/−*^ mice, as demonstrated by VSA studies (Figure 8). This finding aligns with our unexpected observation that *Entpd1* ^*+/−*^ and *Entpd1* ^*−/−*^ mouse BSM cells lack smooth muscle phenotypic abnormalities (Figures S3 and S4). The efficacy of adenosine A2b receptor activation in restoring abnormal bladder function further underscores the critical role of purinergic signaling in bladder physiology and highlights its potential translational value in LUTS treatment.

In summary, we have demonstrated that ENTPD1 dysregulation leads to profound abnormalities in bladder voiding, urodynamics, and BSM contractility, primarily mediated by disrupted extracellular BSM ATP/ADP/adenosine metabolism and altered purinergic receptor expression. We further revealed that inhibition of downstream P2Y12 and activation of adenosine A2b receptor signaling could partially (P2Y12) or fully (A2b) restore bladder dysfunction in these mice. Our identification of ENTPD1 and associated purinergic molecules in human BSM cells also highlights a potential critical role of ENTPD1 and associated purinergic responses in human bladder function and underscores its translational potential for treatment of LUTS patients.

## Author Contributions

W.Y. conceived and supervised the project, performed VSA, CMG, myography, Western blot, analyzed data, and wrote the manuscript. Z.L. performed VSA, Western blot, histological and immunostaining and imaging, and analyzed data. H.C. performed VSA, Western blot, histological and immunostaining and imaging, and analyzed data. A.W. performed VSA. Western blot, immunostaining and imaging, and analyzed data. W.M. and S.B. performed Western blot and analyzed data. S.C.R. and S.L.A. contributed to the conception of the study, and interpretation of data, and revised the manuscript. All coauthors critically reviewed the manuscript, discussed ideas and results, and contributed to the manuscript. W.Y. is the guarantor of this work and, as such, has full access to all the data in the study and takes responsibility for the integrity of the data and the accuracy of the data analysis.

## Funding

This work was supported by HHS, NIH, NIDDK, Division of Kidney, Urologic, and Hematologic Diseases (R01 DK126674, R01 DK135672).

## Conflicts of Interest

S.C.R. is one of the scientific founders of Purinomia Biotech Inc. and consults for eGenesis, AbbVie, and previously for SynLogic Inc. His interests are reviewed and managed by Beth Israel Deaconess Medical Center in accordance with the conflict‐of‐interest policies of Harvard Medical School. Other authors declare no conflicts of interest.

## Supporting information


**Figure S1:** Expression of purinergic pathway proteins in ENTPD1‐dysregulated BSM cells. Mouse bladder tissues immunostained with antibodies detecting ENTPD1 (A), NT5E (B), P2X1 (C), and ENTPD2 proteins (D) (green), colocalized with DAPI‐stained nuclei (blue). In wild‐type mouse bladder (left panels), these proteins (A, B, and C) exhibit clear BSM membrane localization, whereas in *Entpd1* ^*+/−*^ (middle panel) and *Entpd1* ^*−/−*^ mouse bladders (right panel), expression was undetectable (A) or diminished (BandC). ENTPD2 (D) is expressed in bladder interstitial cells at equivalent levels in wild‐type (left panel) and ENTPD1‐dysregulated bladders (middle and right panels). Scale bars, 50 or 100 μm as indicated.
**Figure S2:** Expression of purinergic pathway proteins in CD39TG mouse bladder. A‐F: Western blots of ENTPD1 (*n* = 9), NT5E (*n* = 9), P2X1 (*n* = 9), ENTPD2 (*n* = 6), ENTPD3 (*n* = 6), and ALPL (*n* = 6) proteins in male wild‐type and CD39TG mouse bladders. β‐actin and GAPDH were normalization controls for quantitated data shown in G‐L. Data are plotted in Box (75% of the data) and whiskers format (minimum to maximum), with the centerline as the median value. Student *t*‐test, *p* values above bars.
**Figure S3:** ENTPD1 dysregulation doesn't alter mouse BSM cellular phenotype in the bladder. A‐D: Western blot of CHRM3 (*n* = 6), αSMA (*n* = 9), SM22 (*n* = 6), and MYH11 (*n* = 6) proteins from male wild‐type, Entpd1+/−, and Entpd1−/− mouse bladders. GAPDH served as normalization control for quantitated data in G‐L. Data are plotted in Box (75% of the data) and whiskers format (minimum to maximum), with the centerline as the median value. Student *t*‐test, *p* values above bars.
**Figure S4:** Expression of BSM biomarkers in CD39TG mouse bladder. A–F: Western blot of CHRM3 (*n* = 6), αSMA (*n* = 9), SM22 (*n* = 6), and MYH11 (*n* = 6) proteins from male wild‐type and CD39TG mouse bladders. GAPDH served as normalization control for quantitated data in G‐L. Data are plotted in Box (75% of the data) and whisker format (minimum to maximum), with the centerline as the median value. Student *t*‐test, *p* values above bars.
**Table S1:** Mouse body weight and bladder weight.
**Table S2:** Female mouse bladder smooth muscle layer thickness (*p < 0.05).
**Table S3:** Antibody information.

## Data Availability

Further information, reagents, and other supporting data in this study are available from the corresponding author upon request. Supporting Data Values are in the supplemental XLS file.
