# Supplementary material for: Dysregulation of CD39/Ectonucleoside Triphosphate Diphosphohydrolase 1 Causes Urinary Bladder Dysfunction with Abnormal Smooth Muscle Contractility
Source: FASEB J. 2026 Mar 27;40(7):e71735. doi: 10.1096/fj.202504341RRR (PMC13023731; doi:10.1096/fj.202504341RRR)
Supplement: Supplementary file 1 — Figure S1: Expression of purinergic pathway proteins in ENTPD1‐dysregulated BSM cells. Mouse bladder tissues immunostained with antibodies detecting ENTPD1 (A), NT5E (B), P2X1 (C), and ENTPD2 proteins (D) (green), colocalized with DAPI‐stained nuclei (blue). In wild‐type mouse bladder (left panels), these proteins (A, B, and C) exhibit clear BSM membrane localization, whereas in Entpd1 +/− (middle panel) and Entpd1 −/− mouse bladders (right panel), expression was undetectable (A) or diminished (BandC). ENTPD2 (D) is expressed in bladder interstitial cells at equivalent levels in wild‐type (left panel) and ENTPD1‐dysregulated bladders (middle and right panels). Scale bars, 50 or 100 μm as indicated. Figure S2: Expression of purinergic pathway proteins in CD39TG mouse bladder. A‐F: Western blots of ENTPD1 (n = 9), NT5E (n = 9), P2X1 (n = 9), ENTPD2 (n = 6), ENTPD3 (n = 6), and ALPL (n = 6) proteins in male wild‐type and CD39TG mouse bladders. β‐actin and GAPDH were normalization controls for quantitated data shown in G‐L. Data are plotted in Box (75% of the data) and whiskers format (minimum to maximum), with the centerline as the median value. Student t‐test, p values above bars. Figure S3: ENTPD1 dysregulation doesn't alter mouse BSM cellular phenotype in the bladder. A‐D: Western blot of CHRM3 (n = 6), αSMA (n = 9), SM22 (n = 6), and MYH11 (n = 6) proteins from male wild‐type, Entpd1+/−, and Entpd1−/− mouse bladders. GAPDH served as normalization control for quantitated data in G‐L. Data are plotted in Box (75% of the data) and whiskers format (minimum to maximum), with the centerline as the median value. Student t‐test, p values above bars. Figure S4: Expression of BSM biomarkers in CD39TG mouse bladder. A–F: Western blot of CHRM3 (n = 6), αSMA (n = 9), SM22 (n = 6), and MYH11 (n = 6) proteins from male wild‐type and CD39TG mouse bladders. GAPDH served as normalization control for quantitated data in G‐L. Data are plotted in Box (75% of the data) and whisker forma [file FSB2-40-e71735-s001.docx]

**Supplemental information**

**Dysregulation of CD39/Ectonucleoside Triphosphate Diphosphohydrolase 1 Causes Urinary Bladder Dysfunction with Abnormal Smooth Muscle Contractility**

Zhaobo Luo^1^, Huan Chen^1^, Ali Wu^1^, Weipu Mao^1^, Sagar Barge^1^, Seth L. Alper^1^, Simon C. Robson^1, 2^, and Weiqun Yu^1^*

Department of ^1^Medicine and ^2^Anesthesia, Beth Israel Deaconess Medical Center and Harvard Medical School, Boston, MA 02215

**Supplemental Figure 1.** **Expression of purinergic pathway proteins in ENTPD1-dysregulated BSM cells.** Mouse bladder tissues immunostained with antibodies detecting ENTPD1 (**A**), NT5E (**B**), P2X1 (**C**), and ENTPD2 proteins (**D**) (green), colocalized with DAPI-stained nuclei (blue). In wild-type mouse bladder (left panels), these proteins (**A**, **B**, and **C**) exhibit clear BSM membrane localization, whereas in *Entpd1^+/-^* (middle panel) and *Entpd1^-/-^* mouse bladders (right panel), expression was undetectable (**A**) or diminished (**B**&**C**). ENTPD2 (**D**) is expressed in bladder interstitial cells at equivalent levels in wild-type (left panel) and ENTPD1-dysregulated bladders (middle and right panels). Scale bars, 50 or 100 μm as indicated.


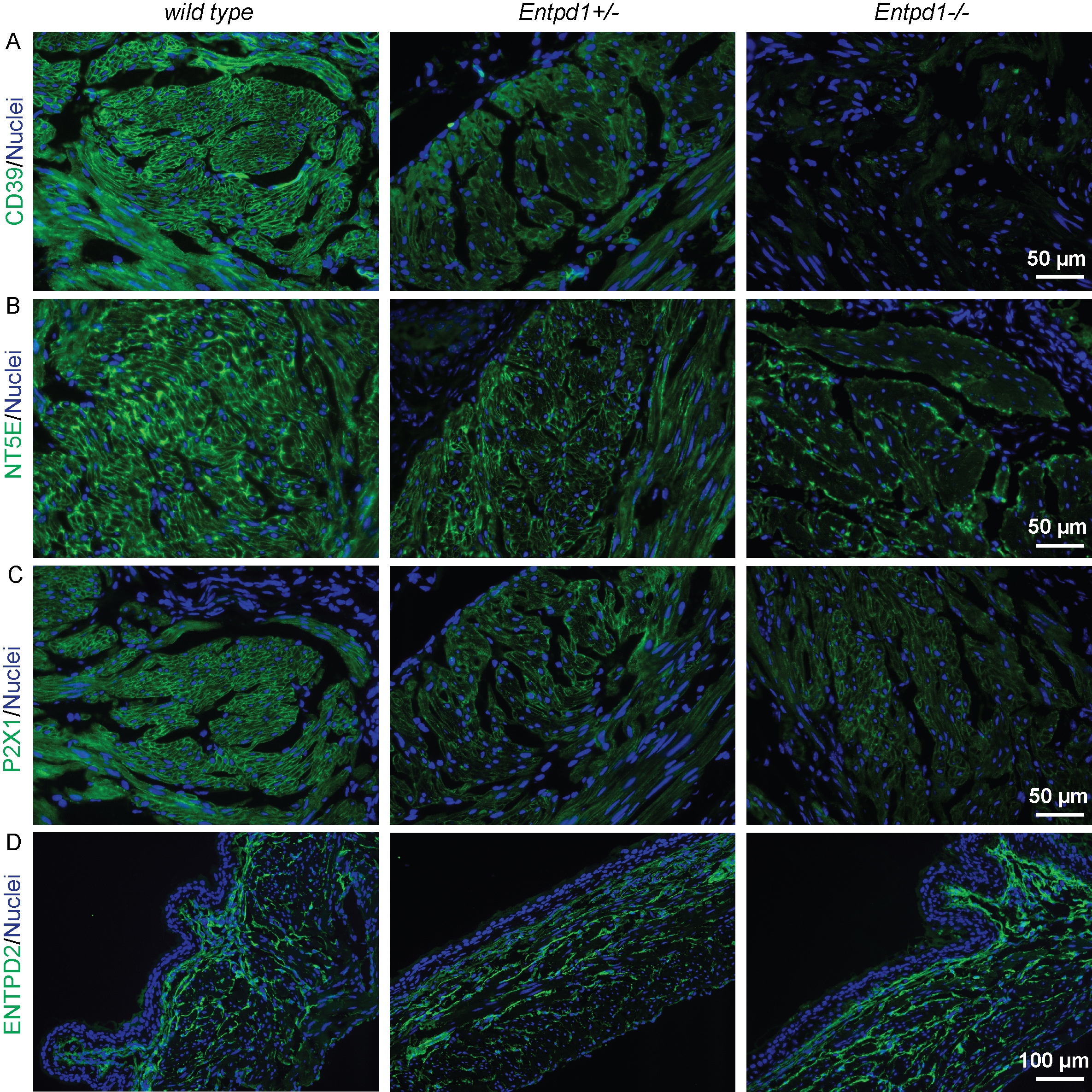


**Supplemental Figure 2. Expression of purinergic pathway proteins in CD39TG mouse bladder.** **A**-**F**: Western blots of ENTPD1 (n=9), NT5E (n=9), P2X1 (n=9), ENTPD2 (n=6), ENTPD3 (n=6), and ALPL (n=6) proteins in male *wild-type* and *CD39TG* mouse bladders. β-actin and GAPDH were normalization controls for quantitated data shown in **G**-**L**. Data are plotted in Box (75% of the data) and whiskers format (minimum to maximum), with the centerline as the median value. Student *t*-test, *P* values above bars.


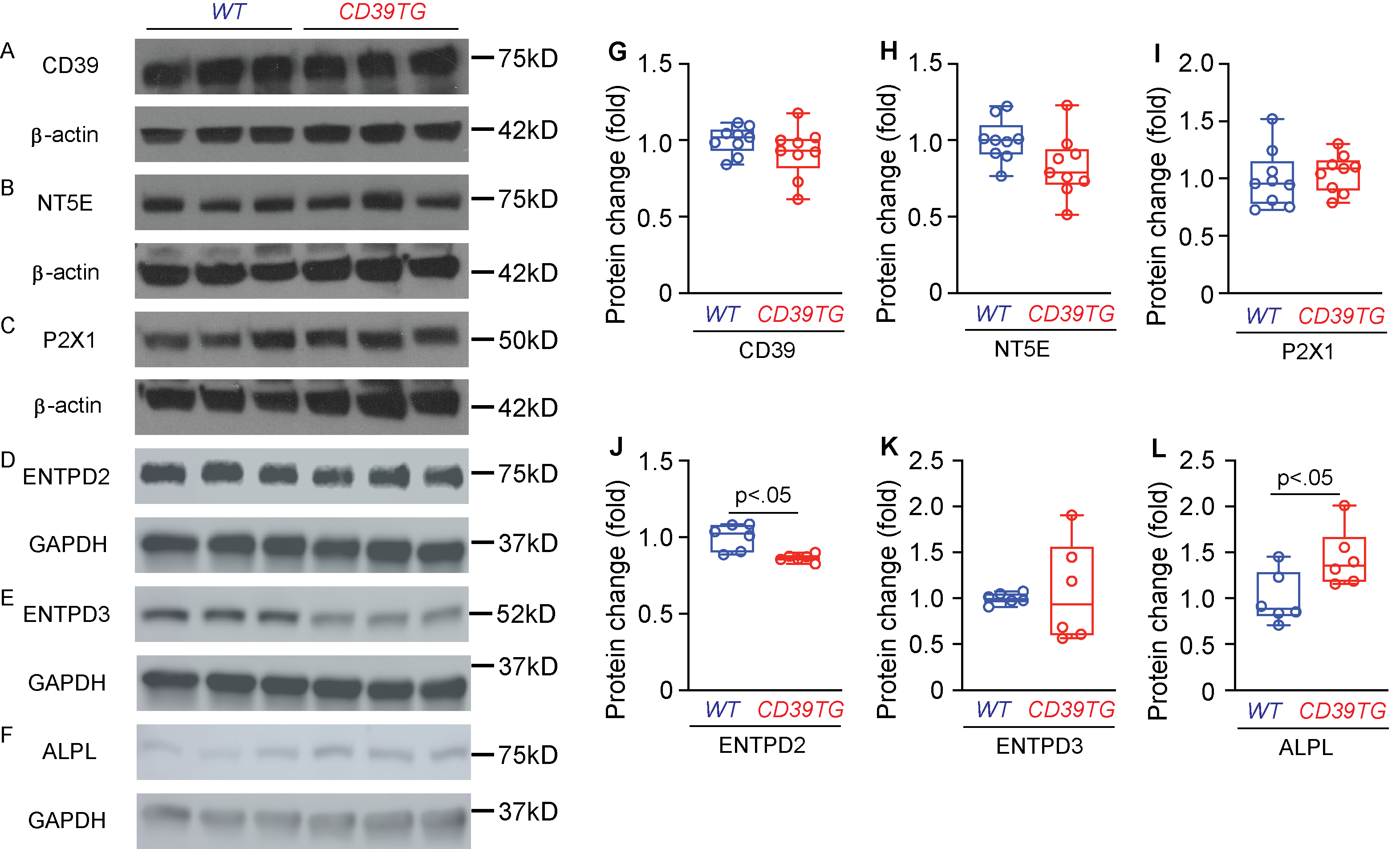


**Supplemental Figure 3. ENTPD1 dysregulation doesn’t alter mouse BSM cellular phenotype in the bladder.** **A**-**D**: Western blot of CHRM3 (n=6), αSMA (n=9), SM22 (n=6), and MYH11 (n=6) proteins from male *wild-type, Entpd1^+/-^*, and *Entpd1^-/-^* mouse bladders. GAPDH served as normalization control for quantitated data in **G**-**L**. Data are plotted in Box (75% of the data) and whiskers format (minimum to maximum), with the centerline as the median value. Student *t*-test, *P* values above bars.


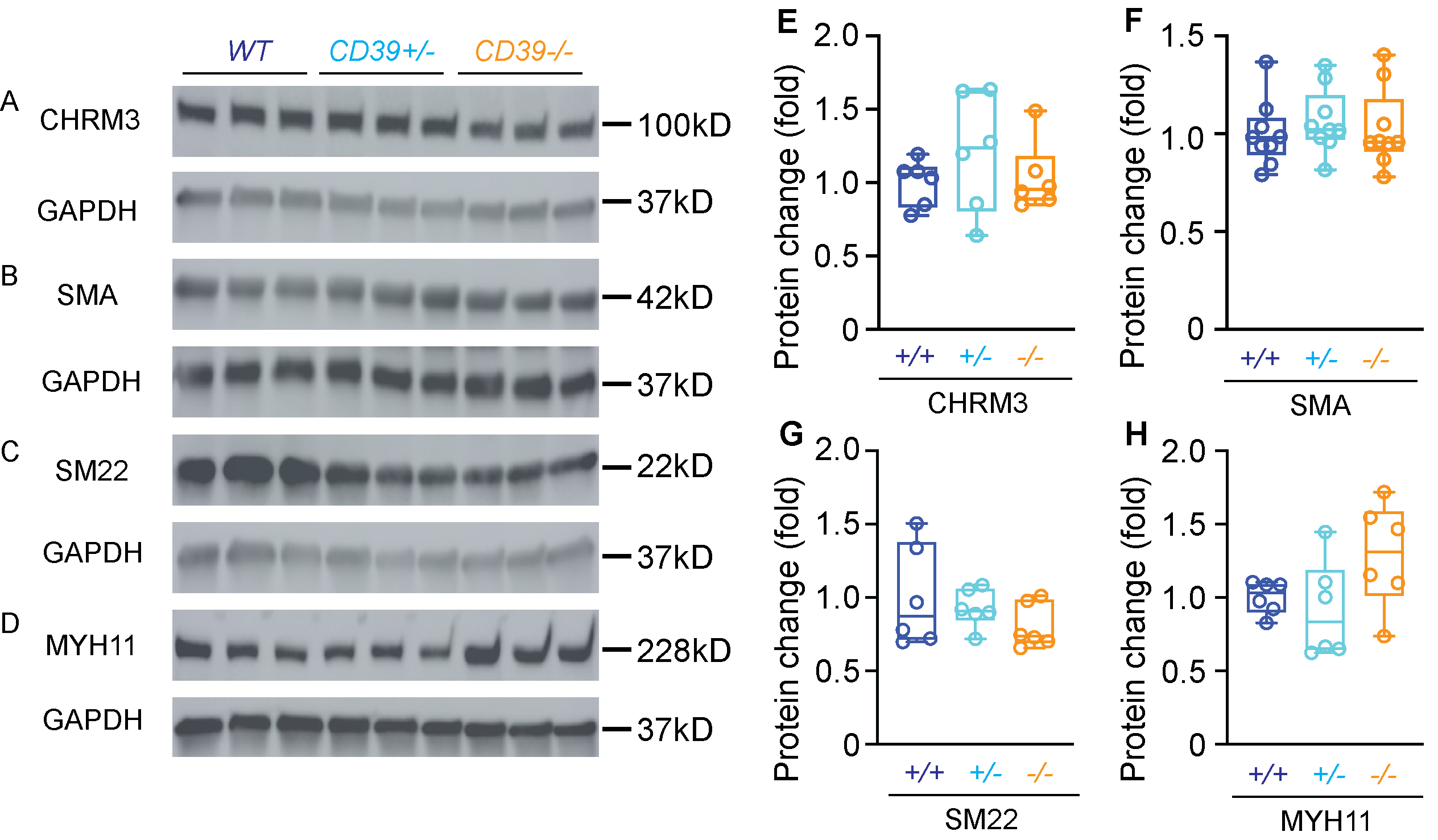


**Supplemental Figure 4. Expression of BSM biomarkers in CD39TG mouse bladder.** **A**-**F**: Western blot of CHRM3 (n=6), αSMA (n=9), SM22 (n=6), and MYH11 (n=6) proteins from male *wild-type* and *CD39TG* mouse bladders. GAPDH served as normalization control for quantitated data in **G**-**L**. Data are plotted in Box (75% of the data) and whisker format (minimum to maximum), with the centerline as the median value. Student *t*-test, *P* values above bars.

Supplemental Table 1. Mouse body weight and bladder weight

|  | Wild type | | CD39TG | | Entpd1+/- | | Entpd1-/- | |
| --- | --- | --- | --- | --- | --- | --- | --- | --- |
|  | Body weight (g) | Bladder weight (mg) | Body weight (g) | Bladder weight (mg) | Body weight (g) | Bladder weight (mg) | Body weight (g) | Bladder weight (mg) |
| Male | 27.5 ± 2.2 (n= 12) | 26.0 ± 1.5 (n=12) | 26.6 ± 1.8 (n=18) | 25.2 ± 2.3 (n=18) | 28.7 ± 2.7 (n=14) | 26.3 ± 1.3 (n=14) | 26.8 ± 1.8 (n=21) | 26.7 ± 2.4 (n=21) |
| Female | 22.4 ± 2.8 (n=14) | 19.3 ± 2.9 (n=14) | 23.0 ± 2.8 (n=19) | 19.5 ± 2.5 (n=19) | 22.2 ± 1.5 (n=12) | 20.5 ± 1.2 (n=12) | 21.3 ± 2.1 (n=14) | 18.5± 1.8 (n=14) |

Supplemental Table 2. Female mouse bladder smooth muscle layer thickness (*: P<0.05)

|  | Wild type (n=7) | CD39TG (n=4) | ENTPD1+/- (n=4) | ENTPD1-/- (n=3) |
| --- | --- | --- | --- | --- |
| BSM thickness (mm) | 0.39±0.07 | 0.42±0.02 | 0.30±0.05 | 0.28±0.01* |

Supplemental Table 3. Antibody information

| Antibody | Company | Catalog | Host | Application |
| --- | --- | --- | --- | --- |
| mENTPD1 | Invitrogen | MA5-32707 | Rabbit | WB |
| mENTPD2 | R&D Systems | AF5797 | Sheep | WB |
| mENTPD3 | Proteintech | 13021-1-AP | Rabbit | WB |
| 5'-Nucleotidase | R&D Systems | MAB44881 | Rat | WB |
| Alkaline Phosphatase | R&D Systems | AF2910 | Goat | WB |
| CHRM3 | Invitrogen | PA585322 | Rabbit | WB |
| P2X1 | Alomone Labs | APR-001 | Rabbit | WB, IF |
| αSMA | Cell Signaling Technologies | 19245S | Rabbit | WB |
| TAGLN | Cell Signaling Technologies | 40471S | Rabbit | WB |
| SMMHC | Proteintech | 21404-1-AP | Rabbit | WB |
| h5'-Nucleotidase | R&D Systems | AF5795 | Sheep | WB, IF |
| hENTPD1 | R&D Systems | MAB43971 | Mouse | WB, IF |
| hENTPD2 | R&D Systems | AF6087 | Sheep | WB |
| GAPDH  β-actin | ABclonal  Cell Signaling | A19056  4967 | Rabbit  Rabbit | WB  WB |
